# M2BP inhibits HIV-1 virion production in a vimentin filaments-dependent manner

**DOI:** 10.1038/srep32736

**Published:** 2016-09-08

**Authors:** Qin Wang, Xiaolin Zhang, Yuling Han, Xinlu Wang, Guangxia Gao

**Affiliations:** 1 CAS Key Laboratory of Infection and Immunity, Institute of Biophysics, Chinese Academy of Sciences, Beijing, 100101, China; 2 University of Chinese Academy of Sciences, Beijing, 100049, China

## Abstract

M2BP (also called 90K) is an interferon-stimulated gene product that is upregulated in HIV-1 infection. A recent study revealed that M2BP reduces the infectivity of HIV-1 by inhibiting the processing of the viral envelope protein. Here we report that in addition to reducing viral infectivity, M2BP inhibits HIV-1 virion production. We provide evidence showing that M2BP inhibits HIV-1 Gag trafficking to the plasma membrane in a vimentin-dependent manner. When vimentin filaments were collapsed by treating cells with acrylamide or by overexpression of a dominant-negative mutant of vimentin, M2BP inhibition of HIV-1 virion production was significantly relieved. We further show that M2BP interacts with both HIV-1 Gag and vimentin and thereby mediates their interactions. We propose that M2BP traps HIV-1 Gag to vimentin filaments to inhibit the transportation of HIV-1 Gag to the plasma membrane. These findings uncover a novel mechanism by which a host antiviral factor inhibits HIV-1 virion production.

The virion particles of HIV-1 encapsidate the RNA genome in a core particle formed from the Gag protein, surrounded by a membrane bilayer containing the viral envelope (Env) protein^1^. Gag alone is able to assemble into virion-like particles that bud out from the producer cells, although efficient production of infectious virus requires other viral proteins^2^. Co-translationally myristylated Gag assembles in the cytoplasm to form a series of intermediate complexes that are transported onto the plasma membrane, associating with the HIV-1 genomic RNA and several cellular factors^3^. On the plasma membrane, Gag further assembles and forms spherical immature capsids that undergo budding^3^.

Type I interferons (IFNs) inhibit HIV-1 replication largely through inducing the expression of a repertoire of host restriction factors^4^,^5^. Restriction factors inhibit the replication of HIV-1 at various steps of the viral life cycle using different mechanisms. A few such antiviral factors have been reported to inhibit the assembly and budding of HIV-1. TRIM22 inhibits HIV-1 virion production through interfering with Gag transportation to the membrane^6^. The phosphodiesterase enzyme CNP inhibits HIV-1 assembly on the plasma membrane^7^. Budding of the virion particles can be inhibited by ISG15^8^ and BST-2, the latter of which is antagonized by the virus encoded Vpu^9^,^10^. Viperin inhibits HIV-1 virion production without affecting the intracellular Gag protein levels, but the underlying mechanism is not yet clear^11^.

Mac-2 binding protein (M2BP, also named 90K, LGALS3BP and BTBD17B), an IFN stimulated gene product, is a glycosylated secreted protein and is detected in the extracellular matrix of several tissues and in the extracellular fluids such as serum and breast milk^12^,^13^,^14^. M2BP was first identified as a tumor-associated antigen^12^,^13^ and reported to be upregulated by both type I and type II IFNs^15^. Intracellular M2BP regulates centriole biogenesis and its overexpression leads to dispersion of pericentriolar material^16^. Elevated serum or tissue levels of M2BP have been observed in some tumors and viral infections including HIV-1 infection^17^,^18^,^19^,^20^,^21^,^22^,^23^,^24^. A recent study showed that overexpression of intracellular M2BP reduces the infectivity of HIV-1 virion particles by decreasing the level of mature HIV-1 Env^25^.

Vimentin (VIM) is a type III intermediate filament protein expressed in undifferentiated and proliferative cells of mesenchymal origin, including leukocytes^26^,^27^. VIM has been reported to regulate cell attachment, migration, signaling, neurite extension and vascularization^26^,^27^. Vimentin filaments can be collapsed by treating cells with acrylamide^28^ or by overexpression of a dominant negative mutant that bears a point mutation in the consensus motif in coil1A (R113C)^29^. In some viral infections, VIM forms cages surrounding viral protein aggregates and the cages are sequestered in aggresomes located at the microtubule organizing center^30^,^31^. VIM has been reported to be required for the trafficking of blue tongue virus to the cell surface^32^. The involvement of vimentin in HIV-1 virion production has not been documented.

Here, we show that in addition to inhibiting HIV-1 Env processing, M2BP inhibits virion production in a vimentin filaments-dependent manner. We provide evidence implicating that M2BP bridges HIV-1 Gag and vimentin filament interactions and thereby interferes with HIV-1 Gag trafficking to the plasma membrane.

## Results

### IFN-induced M2BP expression is required for optimal IFN inhibition of HIV-1 replication in MT4 cells

**Figure 1:**
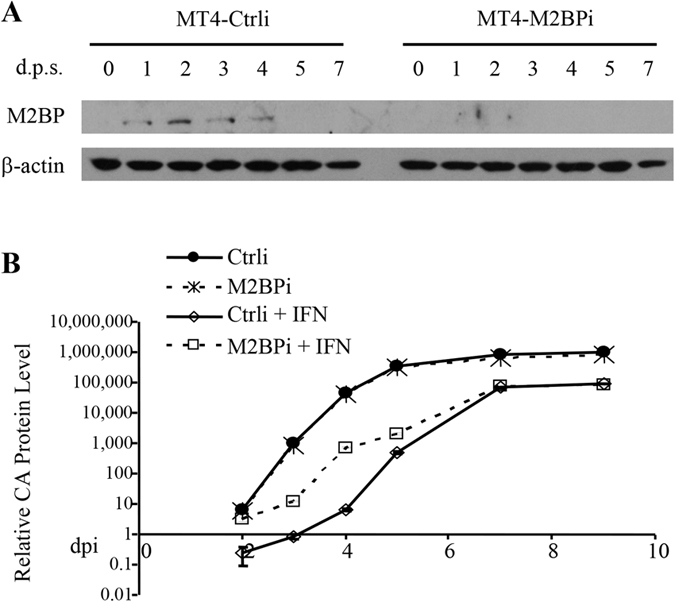
IFN-induced M2BP expression is required for optimal IFN inhibition of HIV-1 replication. A control shRNA (Ctrli) or an shRNA targeting M2BP (M2BPi) was stably expressed in MT4 cells. **(A)** Cells were treated with IFNα-2b and harvested at the time points indicated. Protein levels were measured by Western blotting. d.p.s.: days post stimulation. **(B)** Cells were infected with HIV-1_NL4-3_ virus for 3 h. The virus was removed and IFNα-2b was added to the medium. At the time points indicated, culture supernatants were collected and p24CA levels were measured to monitor viral replication. Data presented are means ± SD of two independent measurements and representative of three independent experiments. d.p.i.: days post infection.

To assess the role of M2BP in IFN inhibition of HIV-1, an shRNA targeting M2BP (M2BPi) was stably expressed in MT4 cells, a cell line derived from CD4+ T cells that support robust HIV-1 replication^33^. The cells were treated with IFNα-2b and M2BP expression levels were analyzed by Western blotting. In the MT4 cells expressing a control shRNA, M2BP expression level was relatively low and was significantly upregulated by IFNα-2b treatment (Fig. 1A). In contrast, in the MT4-M2BPi cells, IFNα-2b treatment barely induced the expression of M2BP (Fig. 1A). These results confirmed that M2BP expression is induced by IFNα-2b and that the shRNA targeting M2BP is effective. The cells were infected with HIV-1_NL4-3_ followed by treatment with IFNα-2b. Viral replication was monitored by measuring the CA (p24) levels in the culture supernatants. IFNα-2b treatment dramatically inhibited HIV-1 replication in the control MT4 cells (Fig. 1B),consistent with the results previously reported^34^. In comparison, in the MT4-M2BPi cells, IFN inhibition of viral replication was significantly reduced (Fig. 1B). These results indicate that IFNα-2b-induced M2BP expression is required for optimal IFNα-2b inhibition of HIV-1 replication in MT4 cells.

### M2BP inhibits both HIV-1 Env processing and virion production

**Figure 2:**
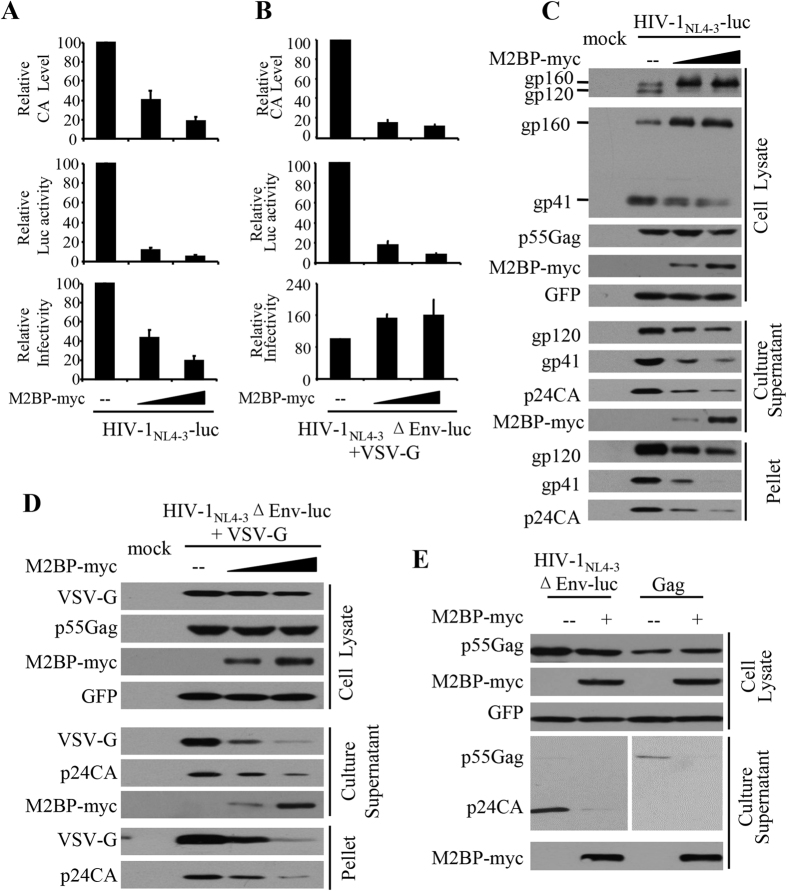
M2BP inhibits both HIV-1 Env processing and virion production. **(A**–**D)** An empty vector (−) or increasing amounts of a plasmid expressing M2BP-myc was transfected into HEK293T cells together with the HIV-1-producing plasmids indicated. A plasmid expressing GFP was included to serve as a control for transfection efficiency and sample handling. **(A,B)** At 48 h posttransfection, p24CA levels in the culture supernatants were measured by ELISA (upper panel). Equal volumes (middle panel) or equal amounts of CA-containing culture supernatants (lower panel) were used to infect HeLa-CD4-CCR5 cells. At 48 h postinfection, luciferase activity was measured in the recipient cells. Relative specific infectivity was defined as the luciferase activity in the recipient cells divided by the amount of the CA-containing virus used to infect the cells. The relative CA protein level, luciferase activity and specific infectivity in the absence of M2BP were set as 100. Data presented are means ± SD of three independent experiments. **(C,D)** The virions in culture supernatants were pelleted by ultracentrifugation. Protein expressions in the producer cells, culture supernatants and virion pellets were analyzed by Western blotting. Mock: mock transfected cells. **(E)** The plasmids indicated were transfected into HEK293T cells with (+) or without (−) a plasmid expressing M2BP. A plasmid expressing GFP was included to serve as a control. At 48 h posttransfection, cells and culture supernatants were harvested for Western blotting analyses.

To understand how M2BP is involved in IFN inhibition of HIV-1 replication, we analyzed the effect of M2BP overexpression on HIV-1 production. Myc-tagged M2BP was transiently expressed in HEK293T cells with the HIV-1 producing plasmid pHIV-1_NL4-3_-luc, a replication-competent HIV-1 proviral vector carrying a luciferase reporter^35^. The produced virus was used to infect recipient HeLa-CD4-CCR5 cells. Overexpression of M2BP reduced the CA levels in the culture supernatants (Fig. 2A, upper panel and Fig. 2C) as well as in the virion pellets (Fig. 2C). However, M2BP had little effect on p55Gag expression in the producer cells (Fig. 2C). When equal volumes of the culture supernatants were used to infect HeLa-CD4-CCR5 cells, the luciferase activity was dramatically reduced in the recipient cells that were infected with the virus produced from the cells overexpressing M2BP (Fig. 2A, middle panel). To evaluate the specific infectivity of the produced virion particles, equal amounts of CA-containing virion particles were used to infect the recipient cells. M2BP overexpression also reduced the relative specific infectivity of the virion particles, which was defined as the luciferase activity expressed in the recipient cells divided by the amount of the virion particles used to infect the cells (Fig. 2A, lower panel). The reduction in the specific infectivity is presumably a result of the reduction in the mature HIV-1 Env protein levels (Fig. 2C), as previously reported^25^. Collectively, these results indicate that overexpression of M2BP inhibits both HIV-1 Env processing and virion production.

The above inhibitory effects of M2BP on HIV-1 virion production and Env processing were analyzed using the replication-competent virus HIV-1_NL4-3_-luc. To further confirm that M2BP overexpression inhibits the production of HIV-1 virion particles, we analyzed the effect of M2BP on the production of vesicular stomatitis virus G (VSV-G)-pseudotyped HIV-1 vector, HIV-1_NL4-3_ΔEnv-luc, in which an engineered stop codon abolishes Env expression^36^. Indeed, overexpression of M2BP also reduced the CA levels in the culture supernatants with little effect on p55Gag expression in the producer cells (Fig. 2B, upper panel and Fig. 2D). M2BP had little effect on the expression of VSV-G in the producer cells (Fig. 2D). Consistently, the specific infectivity of the VSV-G pseudotyped virion particles was little affected (Fig. 2B, middle panel). Notably, M2BP reduced the VSV-G levels in the culture supernatants as well as in the virion pellets (Fig. 2D). A plausible explanation is that VSV-G was carried to the culture supernatant by the virion particles and reduced virion production led to reduced VSV-G levels in the culture supernatants.

In consideration of the fact that HIV-1 Gag protein alone is able to assemble into virion-like particles that undergo budding and release^2^, we analyzed the inhibitory effect of M2BP on the production of the virion-like particles formed by Gag alone. The coding sequence of Gag and the 5′ untranslated region (UTR) were cloned into a mammalian expression vector under the transcriptional control of the CMV promoter. The resulting construct, referred to as pGag, expressed Gag poorly but at a detectable level. Data showed that overexpression of M2BP reduced the Gag levels in the culture supernatants, with little effect on the Gag expression levels in the producer cells (Fig. 2E). These results indicate that HIV-1 Gag alone is responsive to M2BP inhibition of virion production.

To determine whether overexpressed M2BP functions at a level achievable under physiological conditions, increasing amounts of a plasmid expressing M2BP-myc were expressed in HeLa cells. M2BP displayed dose-dependent antiviral activity (Supplementary Fig. 1A). When 20 ng of the plasmid was used, M2BP displayed significant antiviral activity (Supplementary Fig. 1A). M2BP protein levels in HeLa cells and human primary macrophages stimulated with IFNα-2b were analyzed by Western blotting. Downregulation of M2BP with an shRNA reduced the intensity of the band detected by the anti-M2BP antibody, which confirmed the endogenous M2BP band (Supplementary Fig. 1B). The M2BP-myc level expressed from 20ng of the transfected plasmid was below the endogenous M2BP level in HeLa cells and comparable to that in macrophages (Supplementary Fig. 1B). These results indicate exogenous M2BP functions at a level achievable under physiological conditions.

**Figure 3:**
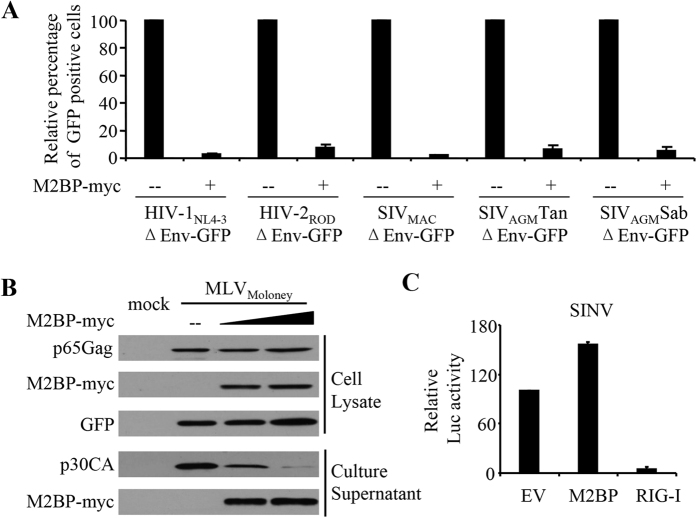
M2BP inhibits the production of MLV and VSV-G pseudotyped lentiviral vectors. **(A)** A plasmid expressing M2BP was transfected into HEK293T cells with plasmids producing the VSV-G pseudotyped lentiviral vectors indicated. At 48 h posttransfection, equal volumes of the culture supernatants were used to infect HeLa-CD4-CCR5 cells. At 48 h postinfection, cells were harvested and analyzed by FACS. The relative number of GFP positive cells in the absence of M2BP was set as 100. Data presented are means ± SD of three independent experiments. **(B)** A plasmid expressing M2BP was transfected into HEK293T cells with an infectious clone of MLV. A plasmid expressing GFP was included to serve as a control for transfection efficiency and sample handling. At 48 h posttransfection, the producer cells and culture supernatants were harvested for Western blotting analyses. Mock: mock transfected cells. **(C)** An empty vector (EV), or a plasmid expressing M2BP or RIG-I was transfected into HEK293T cells. At 36 h posttransfection, cells were infected with SINV-nanoLuc. At 24 h postinfection, the virus-containing culture supernatants were collected and used to infect HeLa-CD4-CCR5 cells for 10 h, followed by luciferase activity measurement. The relative luciferase activity in the absence of M2BP was set as 100. Data presented are means ± SD of three independent experiments.

Overexpression of M2BP also inhibited the production of VSV-G pseudotyped HIV-2_ROD_, SIV_MAC_, SIV_AGM_Tan and SIV_AGM_Sab lentivectors^37^ and replication-competent MLV_Moloney_ virus (Fig. 3A,B). In contrast, M2BP overexpression did not inhibit the replication of Sindbis virus (Fig. 3C).

**Figure 4:**
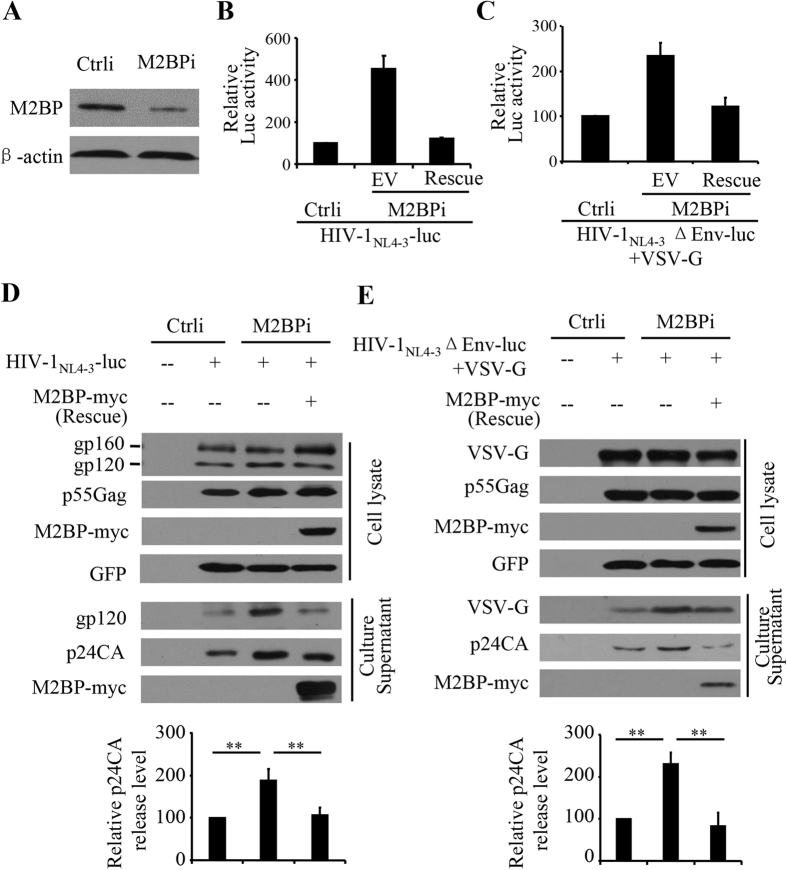
Downregulation of endogenous M2BP enhances HIV-1 virion production. A control shRNA (Ctrli) or an shRNA targeting M2BP (M2BPi) was stably expressed in HEK293T cells. **(A)** M2BP protein levels were measured by Western blotting. **(B–E)** HIV-1-producing plasmids indicated were transiently transfected into cells with (rescue) or without (EV) a rescue M2BP-expressing plasmid. A plasmid expressing GFP was included to serve as a control for transfection efficiency and sample handling. **(B,C)** At 48 h posttransfection, equal volumes of culture supernatants were used to infect HeLa-CD4-CCR5 cells. At 48 h postinfection, luciferase activity was measured. The relative luciferase activity in the cells infected with the virus produced in control cells was set as 100. Data presented are means ± SD of three independent experiments. **(D,E)** The producer cells and culture supernatants were harvested for Western blotting analyses. The intensities of the p24CA bands in culture supernatants and the p55Gag bands in cell lysates were quantified with the Image J software. The p24CA release level was calculated as the p24CA level in the culture supernatant divided by the p55Gag level in the cell lysate. The relative p24CA release level in the absence of M2BP was set as 100. Data presented are means ± SD of three independent experiments. **p < 0.01

To further evaluate the antiviral activity of endogenous M2BP, an shRNA targeting M2BP (M2BPi) was stably expressed in HEK293T cells to downregulate M2BP expression (Fig. 4A and Supplementary Fig. 2). The HIV-1-producing plasmid pHIV-1_NL4-3_-luc was transfected into these cells and the produced virus was collected to infect HeLa-CD4-CCR5 cells. The luciferase activity in the recipient cells served as an indicator of the production of infectious virion particles. Downregulation of endogenous M2BP increased the production of infectious HIV-1_NL4-3_-luc virus by about 5-fold (Fig. 4B). To confirm the specificity of the shRNA targeting M2BP, a rescue M2BP-expressing plasmid that cannot be targeted by the shRNA was co-transfected with the HIV-1-producing plasmid. As expected, expression of the rescue M2BP-expressing plasmid diminished the increase in the virion production (Fig. 4B). Downregulation of endogenous M2BP also increased the luciferase activity in the recipient cells infected with VSV-G-pseudotyped HIV-1_NL4-3_ΔEnv-luc (Fig. 4C), but to a less extent than the effect on HIV-1_NL4-3_-luc (compare Fig. 4B,C). The difference is consistent with the above results that M2BP inhibited only the production of HIV-1_NL4-3_ΔEnv-luc but inhibited both Env processing and virion production of HIV-1_NL4-3_-luc. Indeed, downregulation of endogenous M2BP increased both Env processing in the HIV-1_NL4-3_-luc-producing cells and CA levels in the culture supernatants (Fig. 4D), and increased the CA levels in the culture supernatants of the HIV-1_NL4-3_ΔEnv-lucproducer cells (Fig. 4E). Collectively, these results indicate that M2BP at an endogenous level inhibits HIV-1 Env processing and virion production.

### Mapping the functional domains of M2BP

**Figure 5:**
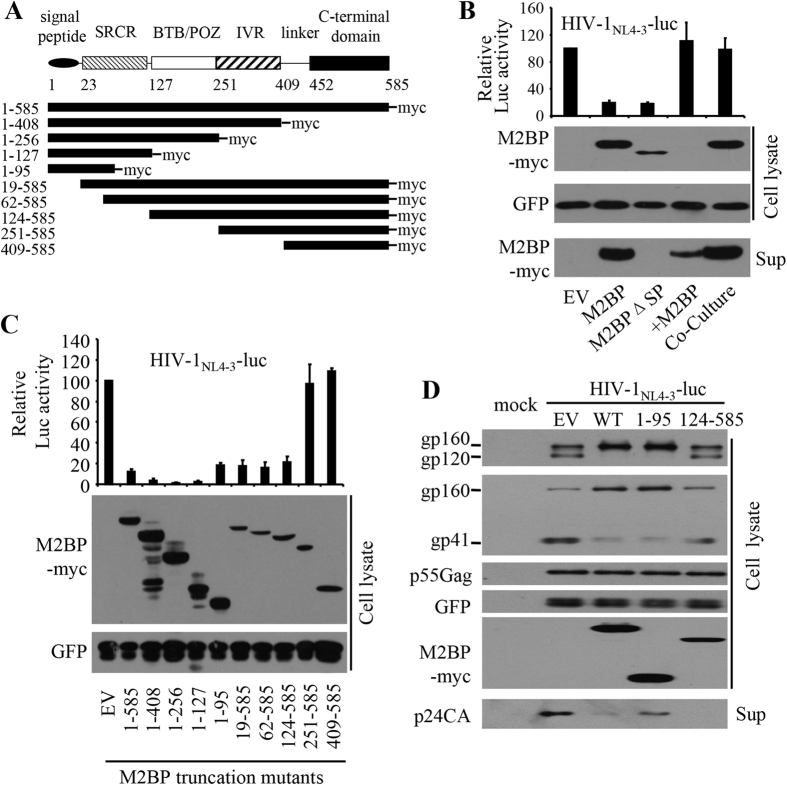
Mapping the functional domains of M2BP. **(A**) Schematic representation of M2BP truncation mutants. **(B)** The HIV-1-producing plasmid indicated was transfected into HEK293T cells with an empty vector (EV), a plasmid expressing M2BP or an M2BP mutant without the signal peptide (M2BPΔSP). In a separate setting, a plasmid expressing M2BP was transiently transfected into HEK293T cells. The M2BP-containing medium was harvested and added to the HIV-1 producing cells (+M2BP), or the M2BP-producing cells were co-cultured with the HIV-1 producing cells (co-culture). The virus containing culture supernatants were collected to infect HeLa-CD4-CCR5 cells. At 48 h postinfection, luciferase activity in the recipient cells was measured (upper panel), and protein levels in the producer cells were analyzed by Western blotting (lower panel). The relative luciferase activity in the absence of M2BP was set as 100. Luciferase activity data presented are means ± SD of three independent experiments, and the Western blotting data are representative of three independent experiments. **(C)** HIV-1-producing plasmid indicated was transfected into HEK293T cells together with a plasmid expressing the M2BP truncation mutants indicated. At 48 h posttransfection, culture supernatants were harvested to infect HeLa-CD4-CCR5 cells and cell lysates were analyzed for expression of the M2BP mutants (lower panel). At 48 h postinfection, luciferase activity was measured in the recipient cells (upper panel). The relative luciferase activity in the absence of M2BP was set as 100. Luciferase activity data presented are means ± SD of three independent experiments, and the Western blotting data are representative of three independent experiments. **(D)** The HIV-1-producing plasmid indicated was transfected into HEK293T cells with a plasmid expressing the M2BP truncation mutants indicated. A plasmid expressing GFP was included to serve as a control. At 48 h posttransfection, cell lysates and culture supernatants were analyzed by Western blotting. The p24CA release level was calculated as described in the legend to Fig. 4D. Data presented are means ± SD of three independent experiments. Mock: mock transfected cells; sup: culture supernatant.

M2BP is a secreted protein composed of a signal peptide, an N-terminal scavenger receptor cysteine-rich (SRCR) domain, a BTB/POZ domain, an IVR domain and a C-terminal domain (Fig. 5A)^38^. The BTB/ POZ domain was previously identified as the dimerization domain^39^. Solid phase assays showed that a fragment spanning the IVR domain and the C-terminal domain contains binding sites for galectin-3, nidogen, and collagens V and VI^39^. To explore whether M2BP works intracellularly or extracellularly, we analyzed the antiviral activity of an M2BP mutant in which the signal peptide was deleted. The mutant, which now was not detected in the culture supernatants, displayed an antiviral activity comparable to that of the wild-type protein (Fig. 5B). These results suggest that M2BP works intracellularly. To substantiate this notion, culture supernatants containing secreted M2BP from transfected HEK293T cells were added to the culture medium of HIV-1_NL4-3_-luc producer cells. No inhibitory effect was observed (Fig. 5B). Furthermore, when the HEK293T cells transfected with a plasmid expressing M2BP were co-cultured with the HIV-1_NL4-3_-luc producer cells, virion production was not affected either (Fig. 5B). These results indicate that M2BP secretion is not required for M2BP inhibition of HIV-1 virion production.

To further map the functional domains of M2BP, a series of M2BP truncation mutants was constructed (Fig. 5A). The mutants were analyzed for their inhibitory activity on the production of HIV-1_NL4-3_-luc. Data showed that deletion from the N-terminus up to amino acid 124 did not have a dramatic effect but further deletion to amino acid 251 abolished the antiviral activity (Fig. 5C). Deletion from the C-terminus up to amino acid 95 did not dramatically affect the antiviral activity of M2BP (Fig. 5C). Notably, the C-terminal truncation mutants appeared to have higher inhibitory activity than the full-length protein. The seemingly higher activity could be accounted for by the higher protein expression levels in this assay. Collectively, these results suggest that there are two functional domains, amino acids 1–95 and amino acids 124–585. The mutants M2BP(1–95) and M2BP(124–585) were further analyzed for their abilities to inhibit Env processing and virion production of HIV-1_NL4-3_-luc. Interestingly, while M2BP(1–95) inhibited Env processing better to some extent, M2BP (124–585) inhibited virion production more efficiently (Fig. 5D). These results suggest that different domains of M2BP are involved in the inhibition of HIV-1 Env processing and virion production.

### Vimentin filaments collapse relieves M2BP inhibition of HIV-1 virion production

**Figure 6:**
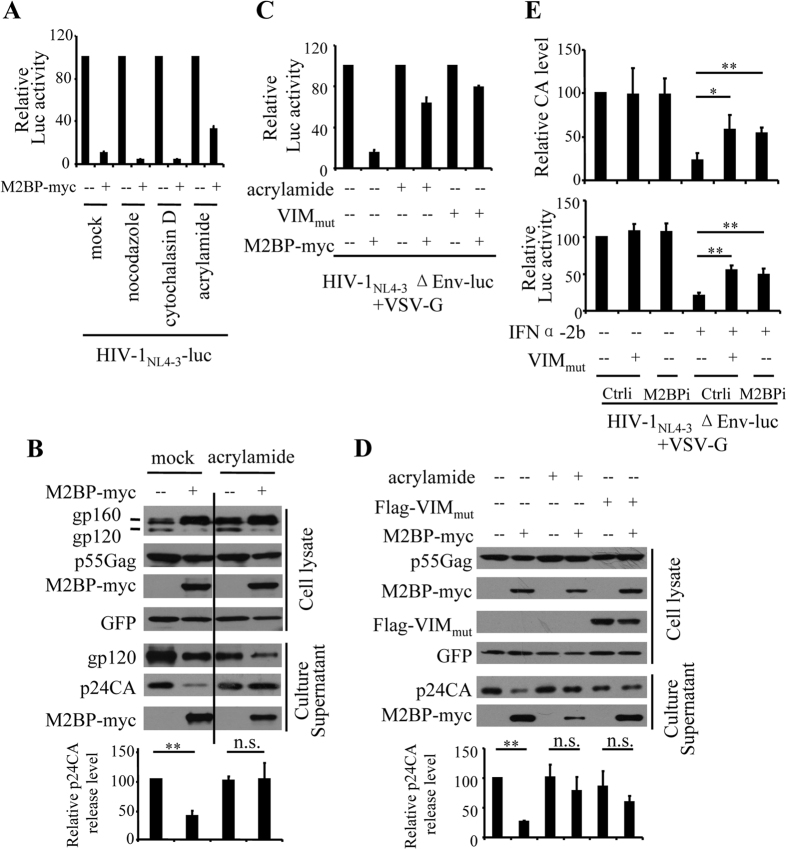
Vimentin filament collapse relieves M2BP inhibition of HIV-1 virion production. **(A–D)** HEK293T cells were transfected with the HIV-1 vector producing plasmids indicated, together with plasmids expressing M2BP and VIM_mut_. At 12 h posttransfection, chemicals indicated were added to the culture media. At 36 h posttransfection, culture supernatants were harvested to infect HeLa-CD4-CCR5 cells or analyzed for protein expressions. At 48 h postinfection, luciferase activity was measured. **(A,C)** The relative luciferase activity in the absence of M2BP was set as 100. Data presented are means ± SD of three independent experiments. **(B,D)** Protein expressions in the cell lysates and culture supernatants. The p24CA release level was calculated as described in the legend to Fig. 4D. Data presented are representative of three independent experiments. **(E)** An empty vector or a plasmid expressing VIM_mut_ was nucleofected into MT4-M2BPi cells together with the HIV-1-producing plasmids indicated. A plasmid expressing firefly luciferase was included to serve as a control for transfection efficiency and sample handling. At 8 h posttransfection, IFNα-2b was added to the culture media. At 48 h posttransfection, p24CA levels in the culture supernatants were measured by ELISA (upper panel). Equal volumes of the culture supernatants were used to infect HEK293T cells. At 48 h postinfection, luciferase activity was measured in the recipient cells (lower panel). Relative CA levels and relative luc activity in the MT4-Ctrli cells without IFN treatment was set as 100. Data presented are means ± SD of three independent experiments. *p < 0.05, **p < 0.01, n.s. p > 0.05

It has been reported that M2BP regulates centriole biogenesis^16^, which participates in the regulation of the morphology of cytoskeletons^40^. This prompted us to ask whether cytoskeletons are involved in M2BP inhibition of HIV-1 virion production. We analyzed the antiviral activity of M2BP in the presence of nocodazole, cytochalasin D and acrylamide, which collapses microtubules, microfilaments and some intermediate filaments, respectively^28^,^41^,^42^. HEK293T cells were transfected with pHIV-1_NL4-3_-luc and treated with the chemicals. The culture supernatants were used to infect recipient cells to evaluate the effects of the chemicals on M2BP inhibition of virion production. Treatment of the cells with acrylamide, but not nocodazole or cytochalasin D, partially relieved the inhibitory effect of M2BP on HIV-1 production (Fig. 6A). Further analyses revealed that treatment of the cells with acrylamide partially relieved M2BP inhibition of virion production without affecting Env processing (Fig. 6B), suggesting that intermediate filaments might be involved in M2BP inhibition of HIV-1 virion production. Notably, in the absence of exogenous M2BP, treatment of acrylamide increased the luciferase activity by about 2-fold (Supplementary Fig. 3), suggesting that collapsing intermediate filaments by acrylamide relieves the inhibitory effect of endogenous M2BP. Only a few types of intermediate filaments have been reported to be collapsed by acrylamide, which include vimentin filaments^28^,^43^,^44^. We speculated that acrylamide might relieve M2BP inhibition of HIV-1 virion production by collapsing vimentin filaments. To test this idea, a vimentin mutant (VIM_mut_), whose overexpression collapses vimentin filaments (Supplementary Fig. 4)^29^, was analyzed for its ability to relieve M2BP inhibition of the production of VSV-G pseudotyped HIV-1_NL4-3_ΔEnv-luc. Indeed, overexpression of VIM_mut_ relieved the inhibitory effect of M2BP on HIV-1 virion production (Fig. 6C,D). To determine whether vimentin filaments are involved in the antiviral function of endogenous M2BP in T cell lines, VIM_mut_ were expressed in the MT4 cells expressing a control shRNA or the shRNA targeting M2BP. IFNα-2b treatment reduced the production of VSV-G-pseudotyped HIV-1 vector in the control cells (Fig. 6E). In comparison, in the MT4-M2BPi cells or in the control cells overexpressing VIM_mut_, IFN inhibition of viral production was significantly reduced (Fig. 6E). Collectively, these results indicate that M2BP inhibits HIV-1 virion production in a vimentin filaments-dependent manner.

### M2BP inhibits HIV-1 Gag localization to the plasma membrane in a vimentin filaments-dependent manner

**Figure 7:**
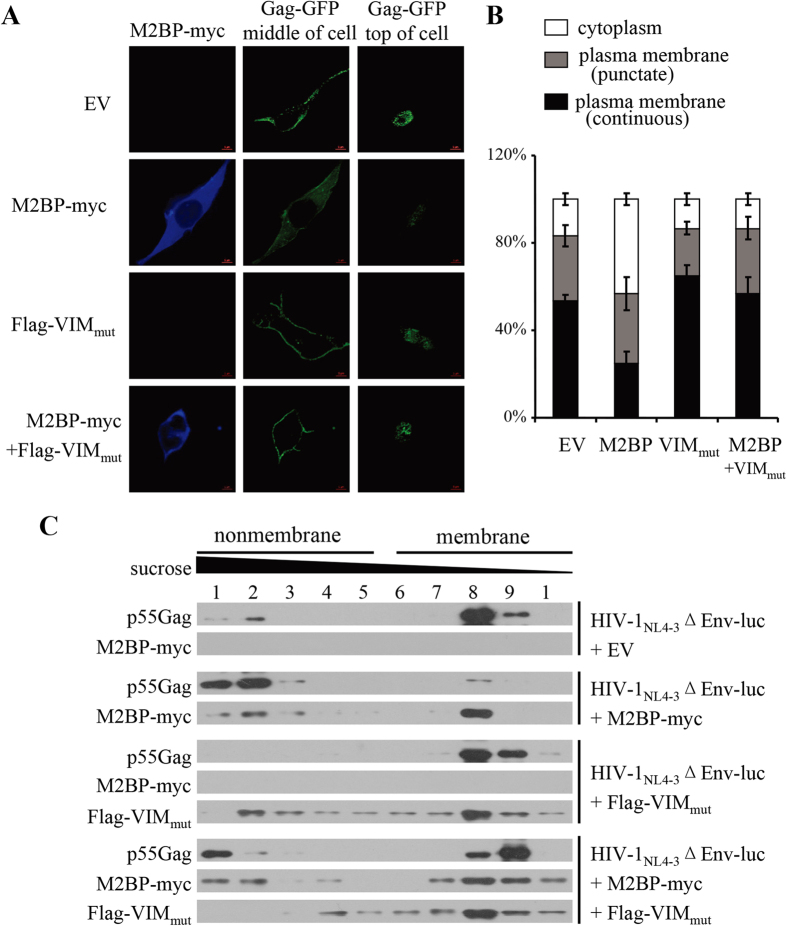
M2BP interferes with HIV-1 Gag localization on the plasma membrane in a vimentin filaments-dependent manner. **(A)** HEK293T cells were transfected with pHIV-1-iGFPΔEnv, together with an empty vector or a plasmid expressing VIM_mut_, with or without a plasmid expressing M2BP. At 36 h posttransfection, cells were fixed in paraformaldehyde. M2BP-myc was stained with anti-myc antibody. Fluorescence images were obtained by confocal fluorescence microscopy. **(B)** Quantification of images in (**A**) from three independent experiments. GFP-positive cells were classified as showing punctate fluorescence or continuous fluorescence at the plasma membrane, or diffuse cytoplasmic fluorescence. **(C)** HEK293T cells were transfected with plasmids expressing the proteins indicated. At 36 h posttransfection, postnuclear supernatants of the cell lysates were analyzed by equilibrium flotation centrifugation, followed by Western blotting analyses. EV: empty vector.

Given that M2BP inhibited HIV-1 virion production with little effect on Gag expression in the producer cells, we next analyzed whether M2BP inhibits HIV-1 Gag localization to the plasma membrane. The HIV-1_NL4-3_ based proviral vector HIV-1-iGFPΔEnv^45^,^46^, in which GFP is inserted between MA and CA, was employed to analyze the localization of Gag by confocal microscopy. In the absence of M2BP, HIV-1 Gag was localized on the plasma membrane (Fig. 7A,B), as reported by others^45^,^46^. Overexpression of M2BP altered Gag localization to be diffuse in the cytoplasm (Fig. 7A,B). When VIM_mut_ was expressed, HIV-1 Gag was now detected on the plasma membrane again (Fig. 7A,B). These results strongly suggest that M2BP interfered with HIV-1 Gag localization on the plasma membrane in a vimentin filaments-dependent manner. To further support this notion, we analyzed whether M2BP affects the association of Gag with the membrane by the membrane flotation assay^47^. Data showed that overexpression of M2BP dramatically reduced Gag association with the membrane (Fig. 7C). When VIM_mut_ was co-expressed, the effect of M2BP on Gag association with the membrane was relieved while overexpression of VIM_mut_ alone had little effect (Fig. 7C). Collectively, these results indicate that M2BP interferes with HIV-1 Gag localization on the plasma membrane in a vimentin filaments-dependent manner.

### Evidence that M2BP mediates the interaction between Gag and VIM

**Figure 8:**
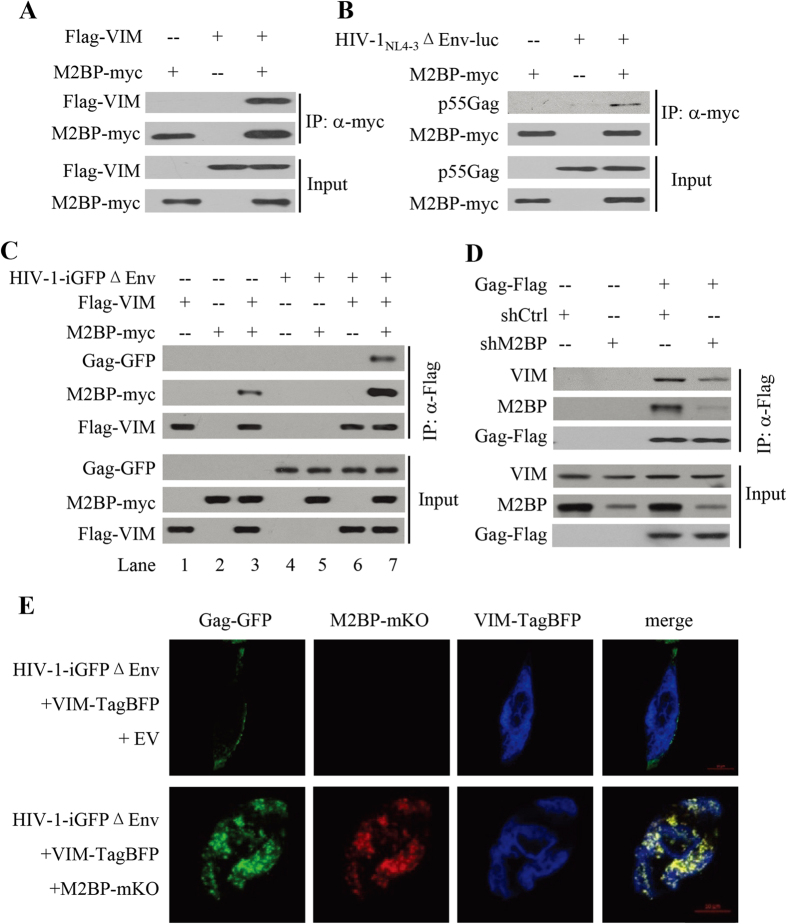
M2BP mediates the interaction between Gag and VIM. **(A–C)** HEK293T cells were transfected with plasmids expressing the proteins indicated. At 48 h posttransfection, cells were lysed and the cell lysates were immunoprecipitated with antibodies indicated, followed by Western blotting analyses. **(D)** Hela cells were transfected with plasmids indicated. At 48 h posttransfection, cells were lysed and the cell lysates were immunoprecipitated with anti-Flag antibody, followed by Western blotting analyses. **(E)** HeLa cells were transfected with pVIM-TagBFP, together with an empty vector or a plasmid expressing M2BP-mKO. At 36 h posttransfection, cells were transfected again with pHIV-1-iGFPΔEnv for 36 h. Cells were fixed in paraformaldehyde. Fluorescence images were obtained by confocal fluorescence microscopy.

To understand how vimentin filaments are involved in M2BP inhibition of HIV-1 localization on the plasma membrane, we analyzed the interactions between M2BP, Gag and VIM. Myc-tagged M2BP was transiently expressed in HEK293T cells together with Flag-tagged VIM or pHIV-1_NL4-3_ΔEnv-luc. M2BP was immunoprecipitated and its interactions with VIM and Gag were assayed by Western blotting. Data showed that M2BP interacted with both VIM (Fig. 8A) and HIV-1 Gag (Fig. 8B). Furthermore, in the absence of M2BP, Gag did not interact with VIM (Fig. 8C, lane 6). However, in the presence of M2BP, Gag interacted with VIM (Fig. 8C, lane 7). Notably, expression of Gag seemed to increase the interaction between M2BP and VIM (Fig. 8C, compare lanes 3 and 7), while overexpression of VIM or VIM_mut_ did not affect the interaction between M2BP and Gag (Supplementary Fig. 5). To further analyze the interaction between Gag, endogenous M2BP and endogenous VIM, pGag-Flag was transfected into HeLa cells and cells were lysed for immunoprecipitation with anti-Flag antibody, followed by Western blotting analyses. The results showed that when Gag-Flag was immunoprecipitated, the endogenous M2BP and endogenous VIM co-immunoprecipitated (Fig. 8D). When the endogenous M2BP was knocked down, the levels of co-immunoprecipitated endogenous VIM were reduced (Fig. 8D). These results suggest that Gag, M2BP and VIM may form a complex coordinated by M2BP.

M2BP (124–585) has been mapped as a functional domain that inhibits the virion production (Fig. 5). We thus examined the interaction between M2BP (124–585), VIM and Gag by immunoprecipitation. Data showed that M2BP (124–585) interacted with VIM and mediated the interaction between VIM and Gag, while the nonfunctional domain M2BP (251–585) only interacted with VIM, but did not mediate the interaction between VIM and Gag (Supplementary Fig. 6). The results suggest that M2BP mediates the interaction between VIM and Gag is necessary for M2BP inhibition of virion production.

To further support this notion, we analyzed the localization of Gag-GFP, M2BP-mKO and VIM-TagBFP. In the absence of M2BP-mKO, Gag-GFP was mainly localized on the plasma membrane while VIM-TagBFP was mainly localized in the cytoplasm (Fig. 8E). However, when M2BP-mKO was overexpressed, co-localization of Gag-GFP, VIM-TagBFP and M2BP-mKO was observed (Fig. 8E). In contrast, no co-localization was observed between M2BP-mKO and GFP (Supplementary Fig. 7), suggesting that the co-localization of Gag-GFP, VIM-TagBFP and M2BP-mKO is specific. To analyze the localization of endogenous M2BP and VIM, we transfected the HIV-1-iGFPΔEnv into HeLa cells and used the anti-M2BP antibody and anti-VIM antibody to stain the endogenous M2BP and VIM, respectively. The results showed that the cytoplasmic Gag-GFP punctate accumulation appeared to colocalize with VIM and M2BP (Supplementary Fig. 8), though not all the cytoplasmic Gag-GFP colocalized with VIM and M2BP. These results further support the notion that M2BP mediates the interaction of Gag with the vimentin filaments.

## Discussion

Type I IFNs inhibit HIV-1 replication by inducing the expression of a variety of host restriction factors^5^. In this report, we show that in MT4 cells, M2BP expression level was upregulated by IFN-α (Fig. 1A) and that downregulation of M2BP significantly reduced IFN inhibition of HIV-1 replication (Fig. 1B). These results indicate that M2BP plays an important role in IFN inhibition of HIV-1 replication in MT4 cells. Overexpression of M2BP also inhibited the production of VSV-G pseudotyped HIV-1_NL4-3_, HIV-2_ROD_, SIV_MAC_, SIV_AGM_Tan and SIV_AGM_Sab vectors and wild-type MLV_Moloney_virus (Fig. 3A,B), suggesting that M2BP inhibition of retrovirus production may be a conserved mechanism.

Recent studies by Lodermeyer V *et al*. showed that overexpression of M2BP reduced the infectivity of HIV-1 virion particles by decreasing the levels of mature HIV-1 Env^25^. Here, we confirmed their observation. In addition, we observed that M2BP inhibited HIV-1 virion production, which was not reported in their studies. However, a close look at the data in their studies revealed that downregulation of M2BP in macrophages also increased the CA levels in the culture supernatants, just to a less extent than that reported here^25^.

Analysis of the M2BP truncation mutants revealed that M2BP(124–585) inhibited virion production more efficiently than M2BP(1–95), while M2BP(1–95) inhibited Env processing better than M2BP(124–585) (Fig. 5D). These results suggest that the mechanisms for M2BP inhibition of HIV-1 virion production and Env processing may be distinct from each other. Consistent with this notion, collapsing vimentin filaments by treating the cells with acrylamide relieved the inhibitory effect of M2BP on HIV-1 virion production without affecting M2BP inhibition of HIV-1 Env processing (Fig. 6C).

Cytoskeletons have been reported to be involved in HIV-1 virion production. Actin and actin-binding proteins were found in HIV-1 virions^48^,^49^, and immunoprecipitation assays revealed that actin interacts with the NC domain of HIV-1 Gag^50^,^51^. Downregulation of endogenous Filamin A or LIMK1, proteins that regulates microfilaments dynamics, or KIF4, a microtubule-based motor protein, interfered with Gag trafficking and led to a decrease in HIV-1 virion production^52^,^53^,^54^. Treatment of cells with nocodazole and cytochalasin D, chemicals that disrupt microtubules and microfilaments, respectively, reduced HIV-1 virion production^55^,^56^. However, the involvement of intermediate filaments in HIV-1 virion production has not been documented. In the present study, we provide evidence showing that vimentin filaments participated in M2BP inhibition of HIV-1 virion production. Treatment of the producer cells with acrylamide or overexpression of a VIM mutant did not generally affect virion production but relieved M2BP inhibition of virion production (Fig. 6). We further show that M2BP interacted with both Gag and VIM and mediated the interaction between Gag and VIM (Fig. 8). Based on these results, we propose a working model for M2BP inhibition of HIV-1 virion production: M2BP interacts with both HIV-1 Gag and VIM, and thereby traps HIV-1 Gag to the vimentin filaments to inhibit HIV-1 Gag trafficking to the plasma membrane. We notice that most of the data supporting this model were obtained by overexpression of the proteins and thus are not very conclusive. Nonetheless, these data add up to be supportive to the model. Further investigation is needed to prove the model.

## Methods

### Plasmids

The plasmid pHIV-1_NL4-3_-luc was kindly provided by Dr. Yonghui Zheng, Michigan State University. The plasmid pHIV-1_NL4-3_ΔEnv-luc was obtained from Dr. Nathaniel Landau through the National Institutes of Health, AIDS Research and Reference Reagent Program. The plasmid pHIV-1-iGFPΔEnv was kindly provided by Dr. Benjamin K. Chen, Mount Sinai School of Medicine. The plasmid pHIV-1_NL4-3_ΔEnv-GFP and pHIV-1_NL4-3_ΔEnv-nanoluc were modified from pHIV-1_NL4-3_ΔEnv-luc by replacing the luciferase coding sequence (CDS) with GFP CDS or nanoluc CDS. The plasmid pGag and pGag-Flag were generated by cloning the 5′UTR and CDS of Gag, PCR-amplified from pHIV-1_NL4-3_ΔEnv-luc, into pcDNA4/TO/myc-HisB (Invitrogen) with or without a double Flag-tag at the C-terminus. The VSV-G-expressing plasmid pVSVG and MLV Gag-pol-expressing plasmid pHIT60 have been described previously^57^.

M2BP and VIM cDNAs were purchased from Sanying Biotechnology (Wuhan, China). The coding sequence of M2BP was cloned into the protein expression vector pLPCX (Clontech) with a triple myc-tag at the C-terminus. To generate constructs expressing M2BP truncation mutants, fragments of the coding sequences were PCR-amplified and cloned into pLPCX with a triple myc-tag at the C-terminus. To generate constructs expressing M2BP-mKO, the coding sequence of mKO was in frame cloned into pLPCX-M2BP. The plasmid expressing VIM_mut_, in which R113 was mutated to a cysteine, was generated by overlapping PCR and cloning into the protein expression vectors pCMV-HF^58^. To generate constructs expressing VIM-TagBFP, the coding sequence of TagBFP was cloned in frame into pCMV-HF-VIM.

The RNAi targeting M2BP was from Thermo Scientific (siGENOME SMARTpool siRNA D-008016-01, LGALS3BP). The shRNA targeting M2BP and a control shRNA (5′-GCGCGCTTTGTAGGATTCGTT-3′) were prepared by annealing pairs of oligonucleotides and cloning into pSuper-Retro-Puro (OligoEngine). To generate a rescue M2BP-expressing construct that cannot be targeted by the shRNA, eight silent mutations were introduced into the target sequence.

### Cell Culture

HeLa-CD4-CCR5 cells have been described previously^59^. HEK293T (ATCC), HeLa (ATCC) andHeLa-CD4-CCR5 cells were maintained in DMEM (Invitrogen) supplemented with 10% FBS (Invitrogen). MT4 cells (ATCC) were maintained in RPMI-1640 (Invitrogen) supplemented with 10% heat-inactivated FBS. To generate HEK293T or MT4 cells stably expressing a control shRNA or an shRNA targeting M2BP, pSuper-Retro-Puro-shCtrl and pSuper-Retro-Puro-shM2BP were transfected into HEK293T cells with pVSVG and pHIT60 to generate transducing retroviral vectors. HEK293T and MT4 cells were transduced, selected with puromycin (Ameresco) and pooled.

To produce HIV-1_NL4-3_-luc and HIV-1_NL4-3_ΔEnv-luc viruses, HEK293T cells were transfected with the producing plasmids. A plasmid expressing renilla luciferase (Promega) was included to serve as a control for transfection efficiency and sample handling. The culture supernatants were used to infect recipient HeLa-CD4-CCR5 cells. Firefly luciferase activity (Promega) in recipient cells was measured and normalized with the renilla luciferase activity in the producer cells.

To purify and concentrate HIV-1 virion particles, virus-containing culture supernatants were ultracentrifugated at 25000 rpm for 2 h using the Hitachi P40ST rotor with a 25% sucrose cushion.

To assay HIV-1 replication in MT4 cells, 0.5 × 10^5^ cells were infected with HIV-1_NL4-3_ containing 0.1 pg of CA. At 3 h postinfection, the infection medium was replaced with fresh medium and IFNα-2b (ProSpec) was added to a final concentration of 10^3 ^U/ml. Culture supernatants were collected at various time points and CA levels were measured using the HIV-Ag/Ab ELISA kit following the manufacturer’s instruction (Wantai, Beijing, China).

To collapse the cytoskeletons, cells were treated with nocodazole (Sigma-Aldrich), cytochalasin D (Stressmarq) or acrylamide (Ameresco) for 24 h at a final concentration of 5 μM, 10 μg/mL or 4 mM, respectively.

### Antibodies

The antibody against β-actin (GSGB-BIO, china, catalogue no. TA-09), myc-specific mouse monoclonal antibody 9E10 (Santa Cruz Biotechnology, catalogue no. SC-40), Flag-specific mouse monoclonal antibody M2 (Sigma-Aldrich, catalogue no. F3165-5MG), Flag-specific rabbit polyclonal antibody (Cell Signaling Technology, catalogue no. 2368P), M2BP-specific antibody (R&D Systems, catalogue no. AF2226), gp120-specific antibody (Abcam, catalogue no. ab21179), gp41-specific antibody (Santa Cruz Biotechnology, catalogue no. sc-57812), anti-mouse IgG (H + L) HRP conjugate (Promega, catalogue no. W4021), anti-rabbit IgG (H + L) TRITC conjugate (GSGB-BIO, china, catalogue no. ZB-0316), anti-rabbit IgG (H + L) HRP conjugate (GSGB-BIO, china, catalogue no. ZB-2301) and anti-mouse IgG (H + L) Cy5 conjugate (Southern Biotech, catalogue no. 1031-15) were commercially available. HIV-1 CA (p24)-specific mouse monoclonal antibody (P5F1)^60^ was a generous gift from Dr. Yong-Tang Zheng, Kunming Institute of Zoology, Chinese Academy of Sciences.

### Coimmunoprecipitation

HEK293T cells were transfected with plasmids expressing HIV-1 Gag or Gag-GFP, myc-tagged M2BP and Flag-tagged VIM. At 48 h posttransfection, cells were lysed in CelLytic™ M Cell Lysis Reagent (Sigma-Aldrich) or RIPA Buffer (140 mM NaCl, 8 mM Na2HPO4, 2 mM NaH2PO4, 1% NP-40, 0.5% sodium deoxycholate, 0.05% sodium dodecyl sulfate, 20 mM iodoacetamide). Clarified cell lysates were mixed with anti-c-myc Agarose Affinity Gel (Sigma-Aldrich) or the Anti-Flag M2 Affinity Gel (Sigma-Aldrich) at 4 °C for 4 h. The resins were washed with TBST (20 mM Tris-HCl pH7.6, 150 mM NaCl, 0.1% Tween-20) five times, and bound proteins were analyzed by Western blotting.

### Membrane flotation analyses

Cell fractionation and equilibrium sucrose density gradient centrifugation assays were modified from those reported previously^47^. Briefly, HEK293T cells were trypsinized, washed with PBS twice and resuspended in TE buffer (10 mM Tris-HCl pH7.5, 4 mM EDTA) supplemented with the complete protease inhibitor cocktail (Roche). Cell suspensions were subjected to sonication in ice water to achieve disruption of more than 90% of cells. After low-speed centrifugation, postnuclear supernatants were adjusted to 150 mM NaCl and mixed with 85.5% (wt/vol) sucrose in TNE buffer (25 mM Tris-HCl pH7.5, 150 mM NaCl, 4 mM EDTA) and placed on the bottom of a centrifuge tube. On top of this postnuclear-supernatant-containing 73% (wt/vol) sucrose mixture was layered 65% (wt/vol) sucrose in TNE and 10% (wt/vol) sucrose in TNE. The gradients were centrifuged at 100,000 g for 18 h at 4 °C. Ten fractions, 1 ml each, were collected from the bottom of the tube for Western blotting analyses.

### Fluorescent staining and confocal microscopy

pHIV-1-iGFPΔEnv was transfected into HEK293T cells with a plasmid expressing M2BP-myc or/and a plasmid expressing VIM_mut_. At 12 h posttransfection, cells were sub-cultured onto coverslips. At 36 h posttransfection, cells were fixed with 4% paraformaldehyde, washed with PBS, permeabilized with 0.2% Triton X-100. After blocking in staining buffer, samples were incubated with the anti-myc antibody, washed with PBS and incubated with Cy5-conjugated anti-mouse IgG. Subsequently, the samples were washed with PBS, and mounted with ProLong Diamond Antifade Mountant (Life Technologies). The subcellular localizations of the stained proteins were photographed using a Laser Confocal Microscope (Zeiss LSM700).

### Statistical Analysis

Mean values ± SD were calculated from at least three independent experiments unless otherwise indicated, and p values were calculated using the Student’s t-test.

## Additional Information

**How to cite this article**: Wang, Q. *et al*. M2BP inhibits HIV-1 virion production in a vimentin filaments-dependent manner. *Sci. Rep.* **6**, 32736; doi: 10.1038/srep32736 (2016).

## Supplementary Material

Supplementary Information
