# Supplementary material for: M2BP inhibits HIV-1 virion production in a vimentin filaments-dependent manner
Source: Sci Rep. 2016 Sep 8;6:32736. doi: 10.1038/srep32736 (PMC5015019; doi:10.1038/srep32736)
Supplement: Supplementary Information [file srep32736-s1.pdf]

M2BP inhibits HIV-1 virion production in a vimentin filaments-dependent manner.

Qin Wang[1,2], Xiaolin Zhang[1,2], Yuling Han[1,2], Xinlu Wang[1#] and Guangxia Gao[1#]

[1]CAS Key Laboratory of Infection and Immunity, Institute of Biophysics, Chinese Academy of Sciences, Beijing 100101, China; [2]University of Chinese Academy of Sciences, Beijing 100049, China

[#]Corresponding author:

Guangxia Gao

Institute of Biophysics, Chinese Academy of Sciences

15 Datun Road, Chaoyang District

Beijing100101, China

Tel: (86)-10-64888545; E-mail: gaogx@moon.ibp.ac.cn

Xinlu Wang

Institute of Biophysics, Chinese Academy of Sciences

15 Datun Road, Chaoyang District

Beijing100101, China

Tel: (86)-10-64844281; E-mail: wang_xl1978@aliyun.com

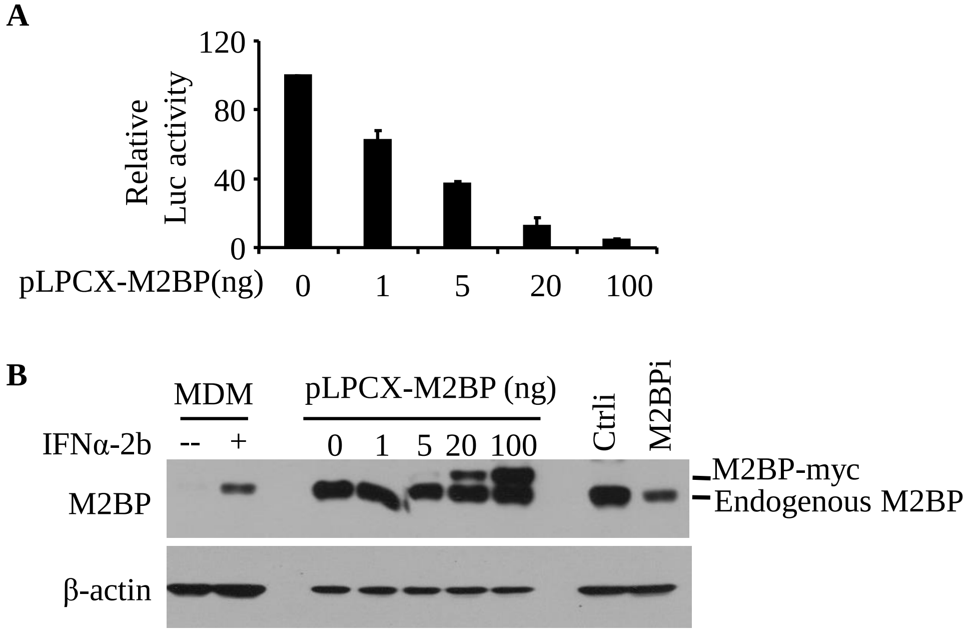

Supplementary Fig. 1 M2BP overexpression in a physiological range inhibits HIV-1 virion production.

(A) Increasing amounts of a plasmid expressing M2BP-myc was transfected into HeLa cells together with the pHIV-1_{NL4-3}ΔEnv-luc and pVSV-G. At 48 h posttransfection, equal volumes of culture supernatants were used to infect HEK293T cells. At 48 h postinfection, luciferase activity was measured. The relative luciferase activity in the absence of M2BP were set as 100. Data presented are means ± SD of three independent experiments. (B) Monocyte Derived Macrophages (MDM) were treated with IFNα-2b. HeLa cells were transfected with a plasmid expressing a control shRNA (Ctrli) or an shRNA targeting M2BP (M2BPi). The same number of macrophages, the shRNA-expressing HeLa cells and the cells in (A) were lysed and subjected to Western blotting analyses.

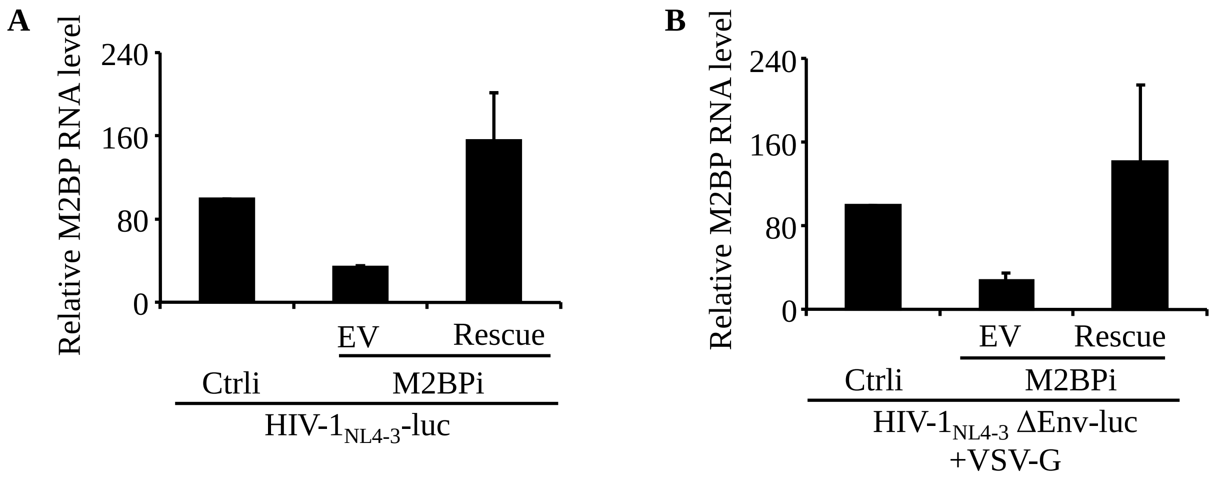

Supplementary Fig. 2 The shRNA targeting M2BP effectively downregulates the M2BP mRNA in HEK293T cells.

(A) and (B) the mRNA of the samples in Fig.4 B and C were isolated for Realtime PCR analyses, respectively.

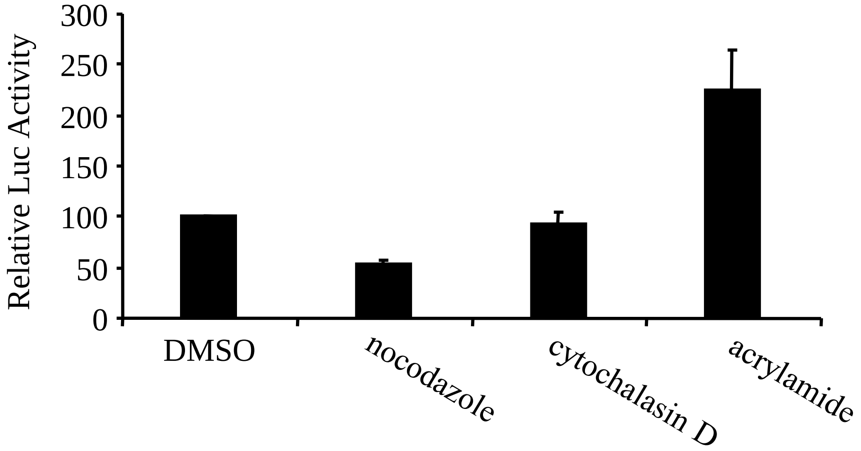

Supplementary Fig. 3 Effects of nocodazole, cytochalasin D and acrylamide on virus production.
Then viron productions from samples without exogenous M2BP-myc (Fig.6A)were selected for comparison. The relative luciferase activity in absence of the chemicals was set as 100. Data presented are means ± SD of three independent experiments.

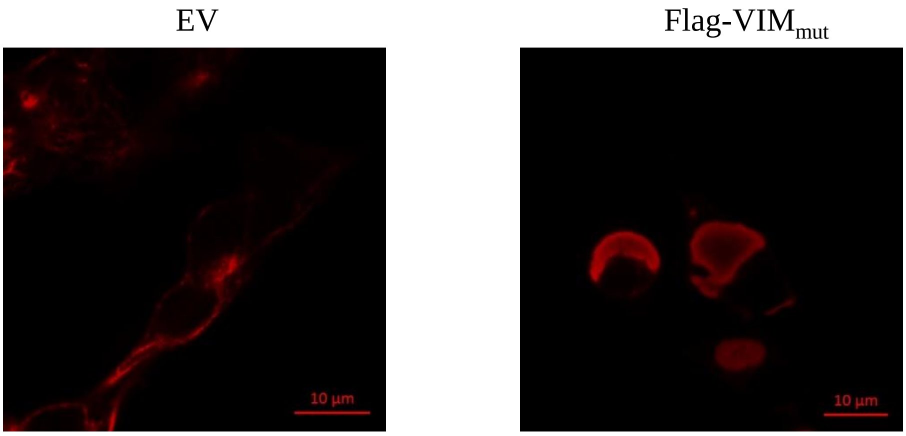

Supplementary Fig. 4 VIM$_{mut}$ overexpression collapses vimentin filaments.
HEK293T cells were transfected with Flag-VIM$_{mut}$. At 36 h posttransfection, cells were fixed in paraformaldehyde. The anti-VIM antibody were used to stain the VIM. Fluorescence images were obtained by confocal fluorescence microscopy.

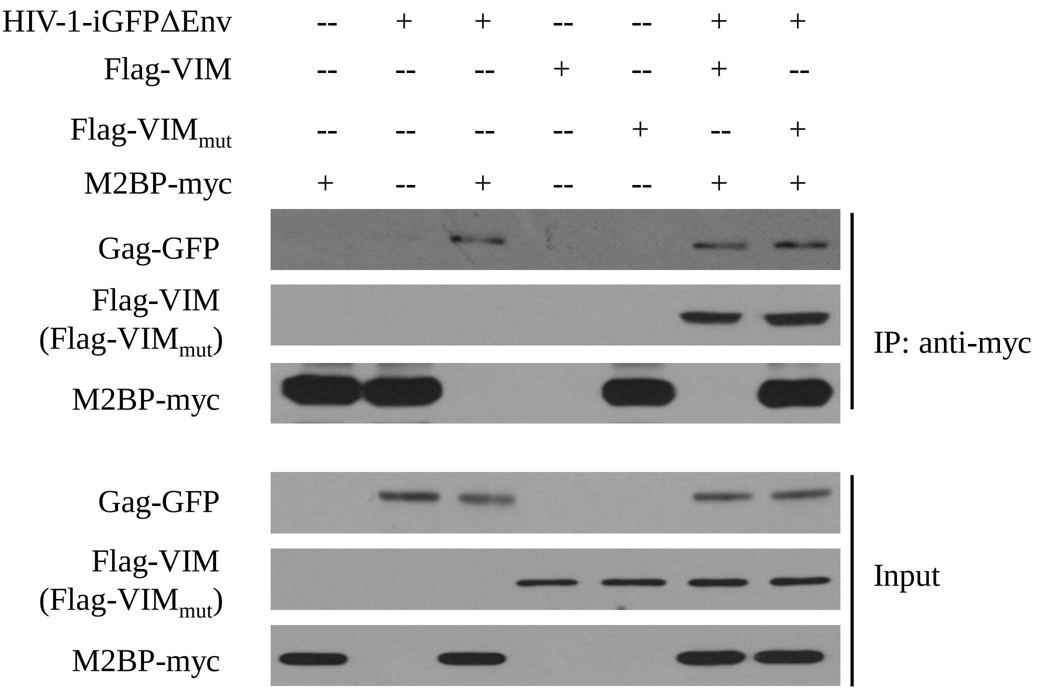

Supplementary Fig. 5 VIM or VIM_{mut} does not affect the interaction of M2BP with Gag.

HEK293T cells were transfected with plasmids expressing the proteins indicated. At 48 h posttransfection, cells were lysed and the cell lysates were immunoprecipitated with antibodies indicated, followed by Western blotting analyses.

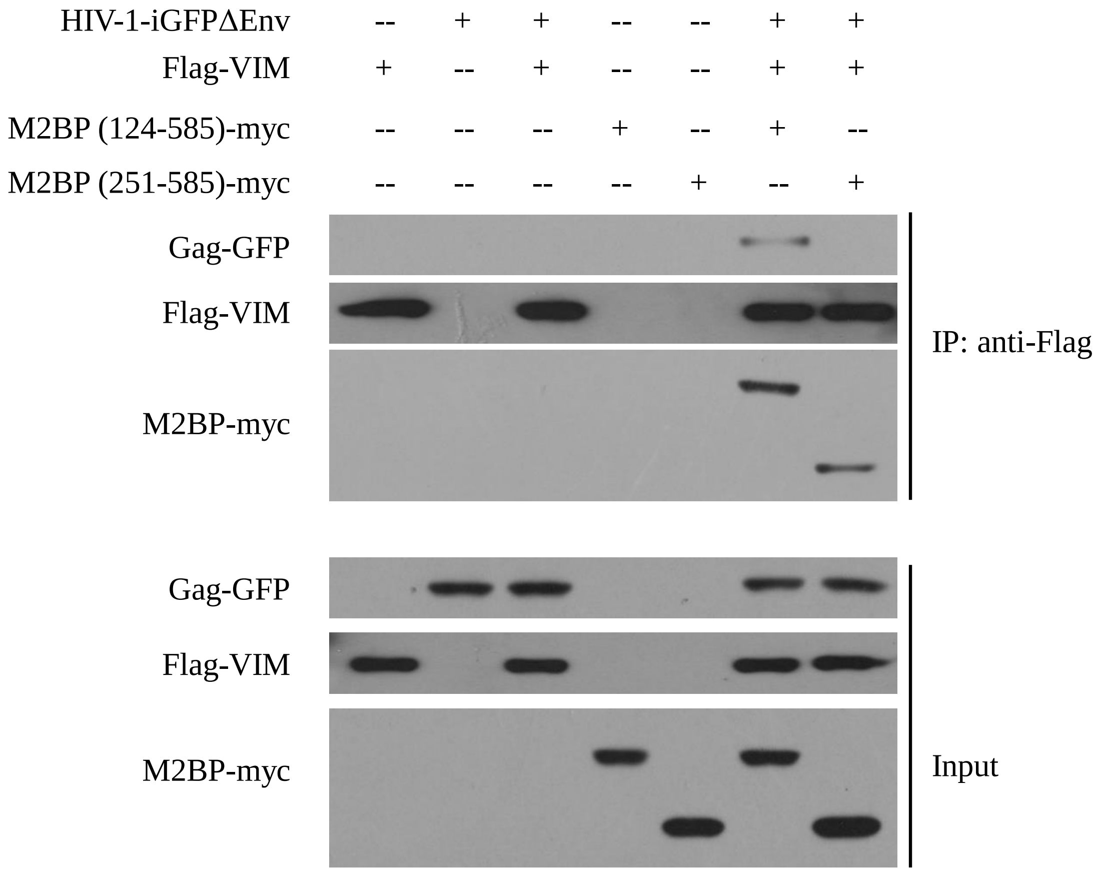

Supplementary Fig. 6 M2BP (124-585) mediates the interaction between VIM and Gag.

HEK293T cells were transfected with plasmids expressing the proteins indicated. At 48 h posttransfection, cells were lysed and the cell lysates were immunoprecipitated with antibodies indicated, followed by Western blotting analyses.

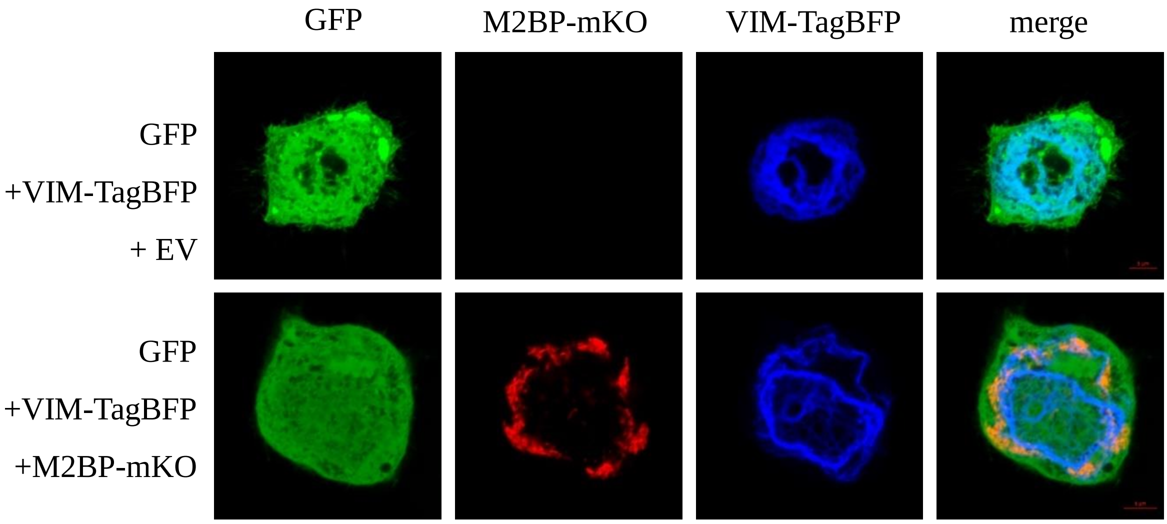

Supplementary Fig. 7 M2BP-mKO has no co-localization with GFP.
HeLa cells were transfected with pVIM-TagBFP, together with an empty vector or a plasmid expressing M2BP-mKO. At 36 h posttransfection, cells were transfected again with pEGFP-N1 for 36 h. Cells were fixed in paraformaldehyde. Fluorescence images were obtained by confocal fluorescence microscopy.

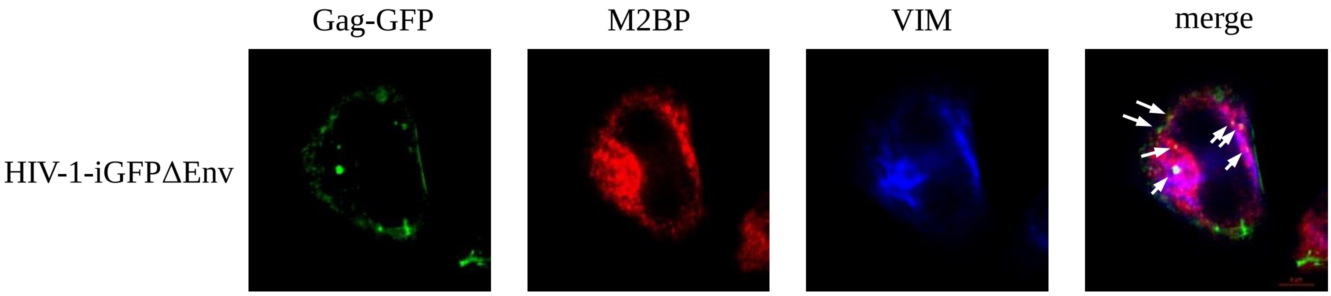

Supplementary Fig. 8 Gag-GFP is colocalized with endogenous M2BP and VIM. HeLa cells were transfected with pHIV-1-iGFPΔEnv. At 36 h posttransfection, cells were fixed in paraformaldehyde. The anti-M2BP antibody and anti-VIM antibody were used to stain the endogenous M2BP and VIM, respectively. Fluorescence images were obtained by confocal fluorescence microscopy. Arrows indicate the positions where the Gag, M2BP and VIM proteins might be co-localized.

Supplementary Methods

Cell Culture

Human monocytes were obtained from peripheral blood mononuclear cells (PBMCs) of a healthy volunteer donor. Briefly, PBMCs were isolated using Lymphoprep (Tian Jin Hao Yang Biological Manufacture). Monocytes were isolated by positive selection using CD14 MicroBeads (Miltenyi Biotec), and further differentiated into macrophages by culture for 7 d in RPMI 1640 supplemented with 10% fetal bovine serum and 50 ng/ml M-CSF (Miltenyi Biotec).

RNA detection

To measure the mRNA levels, RNA was extracted with TRIzol (Invitrogen) and treated with RNase-free DNase (Promega) to avoid plasmid DNA contamination followed by heat inactivation of the enzyme. The RNA was reverse transcribed and detected by Realtime PCR. The sequences of primers for detecting M2BP mRNA and GADPH mRNA are listed below:

M2BP-FP: 5'- GGCAGCAATGTCACCATGAGTG -3';

M2BP -RP: 5'- GTGGAAGCACTTGACTGACGAC -3';

GAPDH-FP: 5'-CCTGGCCAAGGTCATCCATG-3';

GAPDH-RP: 5'-CTCCTTGGAGGCCATGTGGG-3'.

Antibodies

Anti-vimentin rabbit antibody (Cell Signaling Technology, catalogue no. 5741), Donkey anti-Goat IgG (H+L) Secondary Antibody, Alexa Fluor 633 conjugate (Invitrogen, catalogue no. A21082) and Donkey anti-Rabbit IgG (H+L) Secondary

Antibody, Alexa Fluor 568 conjugate (Invitrogen, catalogue no. A10042).
